# Against Personal Ventilator Reallocation

**DOI:** 10.1017/S0963180120000833

**Published:** 2020-10-02

**Authors:** JOEL MICHAEL REYNOLDS, LAURA GUIDRY-GRIMES, KATIE SAVIN

**Keywords:** COVID-19, medical rationing, disability, ventilators, bodily integrity, pandemics

## Abstract

The COVID-19 (Coronavirus disease of 2019) pandemic has led to intense conversations about ventilator allocation and reallocation during a crisis standard of care. Multiple voices in the media and multiple state guidelines mention reallocation as a possibility. Drawing upon a range of neuroscientific, phenomenological, ethical, and sociopolitical considerations, the authors argue that taking away someone’s personal ventilator is a direct assault on their bodily and social integrity. They conclude that personal ventilators should not be part of reallocation pools and that triage protocols should be immediately clarified to explicitly state that personal ventilators will be protected in all cases.

## Introduction

The Coronavirus disease (COVID-19) pandemic has led to intense conversations about ventilator allocation and reallocation during a crisis standard of care (CSC). The ethical value of maximizing lives saved in a pandemic has received widespread support from clinicians and bioethicists for years, so much so that some consider it a fundamental tenet of public health ethics. In the last few months, however, approaches that prioritize maximization of life have received repeated and notable challenges.^1^ One reason for such pushback is the implication that patients who could benefit from a ventilator might have the ventilator withheld or withdrawn if triage officers/teams decide that more patients could be saved by taking it from them.^2^ This reasoning could extend to ventilators outside the hospital setting; if more lives could be saved by taking advantage of chronic-use ventilators in the community, then it would follow that these ventilators should be part of allocation schemas.

This is not a mere academic point. The Food and Drug Administration issued guidance for modifying home- and facility-use ventilators as needed for the COVID-19 pandemic.^3^ In New York, the federal government continually refused to provide needed support and the COVID-19 pandemic overwhelmed hospitals so severely that Governor Cuomo authorized the National Guard to take control of excess community ventilators, announcing his plan in an April briefing that caused some alarm.^4^ As another example, multiple hospitals approached a nursing home in Long Island, requesting access to unused ventilators; the first hospital to make the request received 11 ventilators, leaving the facility with only 5 on hand for current and future residents.^5^ Even outside of New York, healthcare systems globally must find ways to increase supplies and plan for CSC.

Bioethicists, healthcare professionals, and public agencies must pay attention to this concern and clarify promptly: *Are* personal ventilators (PVs) part of the reallocation pool during a pandemic like COVID-19, or not? In this paper, we focus on the primary ethical question that underlies such a decision: *should* they be or not?

We argue that PVs should not be part of reallocation pools and that triage protocols should be immediately clarified and explicitly state that PVs will be protected in all cases. As important as we take our primary argument to be, we take the issues raised by personal ventilator reallocation (PVR) to have biomedical, ethical, and philosophical import far beyond crisis standards of care in the time of COVID-19. First, as we will discuss at length, there is an urgent *moral* need for explicit policies concerning ventilator reallocation at the institutional, state, and federal level. Second, PVR raises crucial *symbolic* issues concerning the historically fraught relationship between the norms of biomedical and public health practices and the needs and wellbeing of the larger disability community.^6^

We begin by analyzing phenomenological, neuroscientific, ethical, and sociopolitical considerations pertaining to the experience of long-term ventilator users, the analysis of which leads us to argue that ventilators should be considered as an *integrated technology*: a technology that is essential to one’s functioning across the life course *and* part of one’s social identity. We then discuss the ways in which integrated technologies such as ventilators have become powerful symbols concerning the worth of disabled lives amidst this political and existential crisis. Heeding the fact that multiple values are at play when CSC are triggered, in the next section, we analyze *four potential scenarios* of PVR during a pandemic as well as the ethical considerations of each. In conclusion, we discuss the larger implications of our argument that reallocating PVs under any circumstances grossly violates bodily and social integrity and conflicts with longstanding biomedical principles and guidelines.

## The Background of PVR Debates

For the purposes of our discussion, the acronym “PV” refers to ventilators that individuals use in their homes, whether that be private homes in a community or in long-term care environments, such as group homes and nursing homes. A person’s financial relationship to these ventilators may differ—for example, one might rent, borrow, or own their ventilators, and this relationship is likely mediated by health insurance. Some bioethicists suggest that whether a ventilator is “rented from a device company” and whether there is “legal ownership” are ethically salient for claims to the ventilator during a pandemic.^7^ For reasons we defend below, we take the nature of the financial relationship to be immaterial to the personal sense of ownership that a ventilator user experiences and the ethical considerations thereof.

The possibility of PVR through a triage process is a source of profound concern for people who rely on ventilators in their everyday life. Alice Wong, a disability activist and a long-term vent user, explains this concern: “Were I to contract coronavirus, I imagine a doctor might read my chart, look at me, and think I’m a waste of their efforts and precious resources that never should have been in shortage to begin with. He might even take my ventilator for other patients who have a better shot at survival than me.”^8^ Although states and individual institutions vary in their PVR policies,^9^ discussion of PVR has been covered in the media and implanted as a concern in disability communities. The introduction of this possibility—that one could go to the hospital to receive acute care services and end up without access to the life-sustaining device that they have constant access to at home—is a valid deterrent to going to the hospital when otherwise necessary. Moreover, silence on an issue during a public health crisis can be decisive; the nature of a crisis is such that, without clear guardrails, options that are ethically problematic can remain on the table in policy discussions and then lead to action. As a result, we contend that there can no longer be neutral, or assumed, defaults in ventilator triage policies.

Our analysis is part of a debate that has existed for over a decade about how chronic-use ventilators should factor into triage decisions during a public health disaster. The 2009 Guidance for Establishing Crisis Standards of Care for Use in Disaster Situations by the Institute of Medicine (now National Academy of Medicine) describes early stages of this debate. According to this report, the World Health Organization at the time argued that “chronic-care patients should be included with all other patients in triage protocols, holding that all must share the sacrifice involved in triage equally,” whereas the Veterans Health Administration “found that viable ethical arguments could support either position,” although they ultimately chose to exclude PVs from reallocation.^10^

Recently in The New York Times, Ari Ne’eman claimed that the 2015 New York triage guidelines would “permit hospitals to take away ventilators from those who use them on an ongoing basis in the community or at a long-term care facility if they seek hospital care.”^11^ Joseph Fins, who served on the Task Force responsible for these guidelines, refuted this characterization of the Task Force’s recommended ventilator reallocation process. The exchange between Ne’eman and Fins underscores the importance of clarity and transparency.^12^ In the past few months, complaints of disability discrimination in triage protocols in Kansas and New York were submitted to the U.S. Department of Health and Human Services Office of Civil Rights, explicitly asking for protection of PVs from reallocation.^13^ Months into the COVID-19 pandemic, the *New England Journal of Medicine* published a perspective piece that advises against PVR in light of disability discrimination complaints, although the ethical reasons for this recommendation are ultimately left unaddressed—the comment is instead a single sentence assertion.^14^ Other bioethicists have left it an open question to what extent PVR should be permitted, raising some doubts about “legitimate” claims and expectations of PV users.^15^

## What is the Lived Experience of a PV?

One of the more established values of medical practice is respect for patient autonomy, including respect for patients’ bodily integrity. Although there are many gray areas in medicine, it is typically taken as a given that infringing on a patient’s bodily integrity should only occur under exceptional circumstances. Yet, what if a patient’s body extends beyond the skin? Is a guide dog part of a patient’s body? What about an implanted cardioverter-defibrillator or a cochlear implant? This is the dilemma faced by people who live with ventilators: will medical professionals view their ventilator as a part of their body and something to be carefully respected or as a mere object they use that can be taken away? It is hard to overstate the personal terror wrought from the suggestion that PVs could be taken away in a triage process, but to fully appreciate *why* this causes such terror requires better understanding the lived experience of long-term ventilator users. If medical providers, health policy experts, and others who play a role in decision-making concerning PVR wish to make informed and ethically defensible decisions, then attention to how long-term ventilator users *experience* their ventilator must be heeded.

Recent neuroscientific studies suggest that the multisensory mechanisms representing peripersonal space do not depend merely upon bodily activity or upon active use of nonbodily items such as tools. On the contrary, peripersonal space is a question of the processing of information resulting from organism–environment interactions. For example, Galli et al., who focus on the use of a wheelchair, argue that a wheelchair “can be conceived as a whole body tool, enabling extended interaction between the person and the environment, thus extending peripersonal space boundaries.”^16^ However, they note that “counter-intuitively, such effect was not induced by active use of the wheelchair in healthy participants who never used a wheelchair before (even if they subjectively reported to “embody” the wheelchair)” (idem). Tellingly, to merely use an object for some discrete period of time is not sufficient for it to become a part of the proprioceptive sensing of an organism.^17^ That is to say, there is a crucial distinction between using an artifact, becoming habituated to using an artifact, and having become habituated to using it to the point of *incorporating* it into one’s experience of one’s body itself.^18^ Seen in this light, long-term ventilator use is not ultimately a question of becoming habituated to the use of a ventilator, but instead of bodily incorporation of the ventilator.

Still, this argument alone does not decide the matter of PVR. We are not primarily concerned with whether a person *senses* or *perceives* their ventilator as a part of their body, but, instead, whether or not such a sensation can act as the foundation of a *moral* claim that removing their ventilator is a harm, including a harm that infringes on their bodily integrity. That is to say, as insightful as phenomenological and biological insights are, they do not, on their own, answer the ethical problem at hand.

To answer that question requires a different analytic toolset. Consider the widely used distinction between curative and assistive technologies. Curative technologies are typically taken to be technological artifacts that contribute to a person’s shift toward comparatively “normal” or “healthy” forms of being, whereas assistive technologies are typically taken to be merely additive and helpful with respect to how one is. In “The Distinction Between Curative and Assistive Technology,” Joseph Stramondo argues that this distinction is untenable if thought in terms of an extensional or intentional definition.^19^ The reason for this is that the *meaning* of the technologies in question does not turn on what the respective concepts are taken to pick out, but instead upon the relationship that a given technology has to one’s “*relational narrative identity* as a member of one of two social groups: disabled people or nondisabled people.”^20^ Stramondo’s claims situate social and dialogical factors relative to politicized identities as definitive—as opposed to biological, phenomenological, and psychological factors.

This is an insightful argument in many respects, but we wonder whether it appropriately extends to the case at hand. Does the permanent use of a ventilator turn, when all is said and done, solely on the *story one tells* about oneself and/or which others tell about one? It seems as though the distinction between therapeutic and assistive technology as tethered to relational narrative identity is insufficient to fully capture the normative dimensions of the use of a technology like a long-term ventilator.

We suggest that the meaning of a ventilator for a long-term ventilator user should instead be construed neither in terms of a therapeutic, nor assistive technology, but instead in terms of what we call an *integrated technology*. More specifically, it is a *corporeally integrated technology*. Although the meaning of a ventilator for a long-term user is certainly determined by their relational narrative identity, as Stramondo’s work rightly suggests, the moral stakes of the relationship between the user and the ventilator is not reducible to that.^21^ This is because without this technology, the person would *die* or would be thrown into a medically dangerous situation. This is part of the reason that ventilators should be treated as prima facie morally distinct from other sorts of similar technologies. Let us unpack this argument further.

In his essay, “Prosthetic Embodiment,” Sean Aas argues that dominant accounts of the moral status of prosthetic or other sorts of bodily extensions fail to motivate expected normative concerns—they do not tell us why we should care about damaging or taking away someone’s prosthetic leg, for example, in the ways that most would expect.^22^ After analyzing why phenomenological, neuroscientific, and biological accounts, such as we are analyzing here, fail to provide such ethical considerations—why they do not tell us how we should distinguish between *morally* meaningful bodily and nonbodily parts—Aas articulates a solution: “to be a body part is to be the sort of thing that ought to be protected, in a certain way, by social practices.”

The question then immediately arises: *which* practices support which sort of social practices in question? “Following arguments from Anita Silvers and other philosophers of disability,” Aas continues, “the thing that social institutions ought to protect, in this bodily way, are those things that are critical to our functioning as equals in our actual social world” (idem). But the philosophical weight is then left on the adjective “critical.” And, furthermore, Aas’ response does not substantively address questions of *how* we adjudicate debates over what is critical and noncritical.

This is understandable for such debates are storied and difficult. Consider the following phenomena: personal computer use, equal representation within a democratic republic, the ability to metabolize the amino acid phenylalanine, access to basic healthcare services, prostheses for lower-limb amputees, and eyeglasses. To determine which of these “are critical to our functioning as equals in our actual social world” and which are not goes to the heart of debates over justice and equity and which—assuming one is operating under real, nonideal conditions—are very hard to answer.

We think, however, that Aas in fact offers a path forward later on in the paper. In a footnote addressing Carter and Palermos’ work on the ethics of extended cognition, he distinguishes between “acts that ought to count as offenses against the person because they are interference with *items that realize cognition*” versus those items that “interfere *in* cognition.” Switching from the issue of cognition to pulmonary functioning, a ventilator, for a long-term user, is not something which merely interferes (positively) in pulmonary function, but which *realizes* livable pulmonary function for them. To bring together both Stramondo’s and Aas’s analyses, then, we can say that what it means to call a ventilator an *integrated technology* is as follows: integrated technologies are essential to one’s functioning, not merely in acute situations, but across one’s future life course, *and* they are part of one’s relational narrative identity.

Someone might object that there could be a long-term ventilator user who resents using their ventilator, who feels alienated from this equipment. Instead of being part of the story they tell about themselves, it is a facet of their life they downplay, ignore, or otherwise diminish. Yet, even in such a case, it *is* a part of their relational narrative identity—they *have* to tell a story about it. Refusing to tell that story or to incorporate various aspects of it does not mean that the story or the parts in question are not constitutive, on at least some integral level, of one’s larger identity—again, even if through refusal or active denial. The ventilator shapes daily interactions and the conditions for living, making a narrative relationship with it mandatory.

Another reason why a ventilator has to be part of one’s relational narrative identity, even if through the mode of refusal, is that it is not simply functionally essential, but it is so in a socially obtrusive way.^23^ Ventilators, based on current technologies, cannot be hidden; on the contrary, ventilators are the sort of thing that one *has to* explain or that are simply *taken as* an aspect of another’s relational identity, even if that persons wishes them not to be so, as discussed above.

It is for all these reasons that a ventilator for a long-term user is in many respects categorically different from other sorts of medical technology. Given these neuroscientific, phenomenological, ethical, and interpersonal reasons, taking a ventilator away from a long-term user is more akin to taking away a part of their body *and* a part of their identity. That is to say, it is more akin to violating both bodily and what we term “social integrity” than it is to taking away a device that someone merely “uses.”

Still, someone might object that this account does not hold for someone who recently started using a long-term ventilator. This objection assumes that the meaning of the ventilator for that person begins at the moment they start using it, but this assumption is misguided. A person who ends up using a long-term ventilator is unlikely to have that option presented to them out of the blue. On the contrary, they were likely on a complicated medical trajectory that led up to their use of such a device. Being discharged with a long-term ventilator is part of a process that most people would have been on for quite some time. Although the harm against someone who has been using a long-term ventilator for a few hours or days would certainly be different in degree than someone who has been using one for 10 or 20 years, we contend that it would not ultimately be different in kind—specifically given the way in which long-term ventilator use constitutes an integrated technology as we have defined that term.

To summarize, what commits us to grant moral status to objects such as ventilators for long-term users, artifacts that are admittedly not prima facie part of one’s “biological” body, is whether or not they are integrated in the sense of both participating in and realizing the essential functions of their biological life *and* the narrative relational identity of their social life. Following Aas, we leave the ultimate meaning of “essential” (or, as he terms it, “critical”) undefined in any technical sense and following Stramondo, we leave the ultimate meaning of “identity” similarly undefined in any technical sense, but we do so because we think that further specifications of those terms do not undermine our central argument. To put things in an overly simplistic manner, it is neither up for debate that *the ability to breathe is essential* for human organisms, nor that extrinsic, socially conspicuous devices affording such essential abilities will become *a part of the story* one and others tells about oneself.

The research we have engaged and the arguments we have put forward lead us to the following conclusion: *taking away someone’s ventilator is a direct assault on their bodily and social integrity*. It is morally akin to taking away a part of their physical body and a part of their social identity. Respect for patient autonomy as well as bodily and social integrity demands that PVs not be taken from those who use them. Even replacements of ventilator devices—due to conditions where shifting to a different type of ventilator device is medically indicated—should be done in careful consultation with patients.

## Medical Decisionmaking, Symbolism, and Disability

As far as these arguments take us, they do not address an equally important consideration: the public, symbolic importance of debates over and decisions concerning PVR. It is one thing to claim that PVR is wrong from a biomedical or philosophical lens; it is another to appreciate the harms that even suggestions of PVR can cause at the larger level of public discourse and the public imaginary, especially in light of centuries of disregard, disparagement, and far worse forms of treatment of numerous communities of people with disabilities both in the United States and abroad.^24^

In this light, it is no surprise that ventilators have become a focal point of resource triage debates and advocacy in many disability communities and have in many ways taken on symbolic meaning amounting to the perception of one’s social worth—whether people are “worth saving” and whether they live “lives worth living,” to invoke the language of the Third Reich’s T4 program.^25^ As horror stories emerged from Italy about ventilator shortages and the painful act of deciding who would or would not receive a ventilator were recounted, the prospect of triaging ventilators was highly anticipated in the United States. The pandemic has underscored and compounded long-standing health inequities in the United States, especially in Black and Latinx communities, including lack of access to testing and health information; lack of access to routine, primary care, and increased exposure to the virus due to lack of personal protective equipment (PPE) and disproportionate numbers of frontline, “essential” workers.^26^ Still, public discourse and broadly publicized communication among elected officials have continued to highlight not the role of systemic inequality and measures to address it, but instead concerns about supply-side issues such as PPE and ventilator access. The outsized presence of ventilators in the media magnifies their symbolism as the machine that gives access to life when all else is lost. New York Governor Andrew Cuomo declared in mid-March, 2020, “Ventilators are to this war what missiles were to World War II,” adding a touch of patriotism to the state’s efforts to procure ventilators.^27^

The COVID-19 pandemic is not the first time that breathing has taken on greater meaning than its mechanical function. In the case of the brutal murder of unarmed Black man Eric Garner in New York City in 2014, police put Mr. Garner in a choke hold that suffocated him. Videos of the attack recorded his final words, “I cannot breathe,” a phrase which he repeated 11 times as he was choked to death.^28^ Later, when swells of outraged people filled the streets protesting the senseless killing, these three words were repeated as rallying cries, on signs, and as a protest hashtag on social media. Again in 2020, when Minneapolis police suffocated another unarmed Black man, George Floyd, the scene was recorded by video and his last words reverberated across the nation: “I cannot breathe.”^29^ The words became a rallying cry again, representing the systematic denial of breath and therefore life.

Air, lungs, breathing and, now, ventilators have long been symbols for essential life and hope. Once used as a means of determining death, the ability to breathe is a fundamental part of human life.^30^ Although biomedical advancement from the iron lung to the ventilator has provided alternative mechanisms for exchanging oxygen and carbon dioxide, one’s capacity to breathe is so fundamental to life it remains one indicator for brain death.^31^ In Sontag’s discussion of the metaphors associated with tuberculosis, she suggests a “disease of the lung” is a “disease of the soul,” so that even in its metaphoric form, the organ responsible for breathing is all-encompassing.^32^

Thus, any discussion of ventilators is inevitably going to provoke strong reactions. As this pandemic has exposed deep societal inequities, discussions of triage protocol related to social identities, such as that of disability, may seem like a referendum on the worth of the lives of these groups. Discourse throughout this pandemic has continually rendered disabled communities as *other*, and also often as *lesser*, than the nondisabled, working-age adult. At the start of the pandemic, news reports repeatedly assured listeners that “only older people and people with underlying conditions” have suffered critical illness and deaths from COVID-19. In response, many disabled activists wrote critiques with sarcastic comments such as, “Do they not know we can read?”^33^

Similarly, the discussions about PVR struck fear into long-term ventilator users and their loved ones that rippled across disability communities. The fact that PVs were ever a point of contention is not likely to be forgotten, especially if triage protocols never explicitly state that PVs are protected from reallocation. In order to work toward establishing trust in healthcare systems—which is an important component of health equity in the present and for the future—the collective trauma of the COVID-19 pandemic needs to be a part of our decisionmaking both today and going forward. For disability communities, this may mean understanding the fears that may arise around accessing basic care and making explicit policies that rebuke particularly charged ideas, such as PVR.

Public health officials, bioethicists, and healthcare providers must contend with the realities of the ventilator as a symbol of life and worth, while not over-focusing on ventilators in a pandemic response to the detriment of increasing access to more basic primary care and PPE. The task for public health messaging is to reframe the meaning of a ventilator as a tool that may help in limited, specific, situations, yet is not in and of itself indicative of quality of care. There are many challenges facing disabled people during this pandemic, and there will be many challenges when the world starts to recover. Now that we have detailed a number of arguments and considerations objecting to the reallocation of PVs, we will turn to address specific scenarios in which the demand for reallocation may arise and explore in far more detail how, in our view, clinicians and providers should respond.

## Cases for Consideration

To better understand how CSC might lead to a PV user not having access to a ventilator during a pandemic like COVID-19, and to make our analysis and recommendations more concrete, we lay out the following four scenarios.

### Scenario 1

Ventilators intended for chronic use outside of the hospital setting could be acquisitioned by hospital systems that are desperate. Local authorities could ask chronic-care facilities to hand over ventilators to shore up dire shortages. This acquisition could focus on *excess* ventilators, that is, those not in use. Alternatively, *all* of the ventilators in these facilities could be considered part of the allocation/reallocation pool, even subjecting residents of chronic-care facilities to a triage process.

Whether retrieving excess ventilators from facilities is ethically permissible would depend on whether this would place undue burden on care facilities that will likely need those ventilators. Without them, facilities may be less able to take new residents (which causes a discharge problem for hospitals), and they may not be able to replace faulty ventilators (creating a precarious threat to current ventilator users in the facility). A reciprocity agreement between the requesting hospital(s) and the facility could help mitigate these concerns. Government agencies should explicitly give chronic care facilities the freedom to opt out of these arrangements, which means giving some protection against negative repercussions to facilities’ funding and public relations if they refuse. If a facility does decide to share their excess ventilators, there should be continual communication and evaluation of the facility’s needs, and there should be clear triggers for returning ventilators back to the facility to prevent time lags that lead to devastating consequences for vulnerable facility residents.

Making *all* chronic-use ventilators part of the reallocation pool has been long-considered ethically impermissible for several reasons. The 2009 IOM/NAM report points out that it would require an unacceptable role reversal for facility caregivers and would cause brutal and unjust harm to persons with disabilities.^34^ The New York State Task Force and Life and Law Report argues: “To triage patients in chronic-care facilities once the Guidelines are implemented may theoretically maximize resources and result in more lives saved, but conflicts with the societal norm of defending vulnerable individuals and communities.” They add, “this approach fails to follow the ethical principle of duty to care and could be construed as taking advantage of a very vulnerable population.”^35^

The COVID-19 pandemic has seen this debate resurface, but we agree with the New York Task Force that PV users in facilities should not be subjected to reallocation for the reasons they suggest, further bolstered by our concerns for bodily and social integrity.^36^ One could counter that PVR in this scenario can be ethically acceptable—perhaps even obligatory—when the resident is in a prolonged vegetative state; this disorder of consciousness might seem to preclude the possibility of the patient ever having a sense of personal ownership over the ventilator. In a public health crisis, therefore, it might seem sensible to reallocate ventilators for this particular population, so more patients in acute need can be saved in the pandemic. We now know, although, that vegetative states are commonly misdiagnosed, and recovery can be possible; permanent vegetative states are no longer even accepted as a nosological category.^37^ Furthermore, the issue of the protection and medical care of people in such states, however, medically defined, goes to the heart of disability rights.^38^ Safeguarding the rights to care and recovery of this vulnerable population is therefore still a fundamental obligation of healthcare professionals and caregiving facilities, and our concerns about bodily and social integrity remain.

### Scenario 2

Whenever a patient comes into the hospital with their PV, the patient might no longer be allowed to “lay claim” to that ventilator. The ventilator becomes part of the allocation pool for the public good, and the patient might not have their ventilator returned to them.

Objections to any such policy are justified.^39^ This would be akin to seizing someone’s vital organ for public use, for all the reasons we have detailed. This leaves people who are dependent on their ventilator little choice but to avoid the hospital at all costs, especially if the triage criteria mean that they might not receive ventilator support during their hospitalization. Furthermore, this scenario would likely cause enormous moral distress for clinicians. To our knowledge, no hospital is considering commandeering PVs, even under CSC. Given the immense fear surrounding this possibility, *hospitals should immediately allay these concerns through policies that explicitly eliminate this possibility*.

### Scenario 3

Someone who relies on a PV for chronic care could be admitted to the hospital. Hospital-based healthcare workers are generally not trained to use home ventilator equipment, so they may want to switch out the PV for a hospital ventilator. The hospital’s ventilator may be functionally equivalent to the home ventilator and the patient’s respiratory support adequately provided by either the PV or the hospital ventilator.

The patient should be consulted about their preferences regarding switching out ventilators in this scenario, given pervasive fears about PV confiscation under CSC. Especially if the type of ventilator in question is scarce, the default should be to work with the patient’s own ventilator. This would be a departure of standard practice, since usually patients do not use their own equipment when they enter the hospital setting, even if they are functionally comparable. If the hospital cannot accommodate continued use of the PV for that particular patient, the PV should be placed in protected storage; even if the PV could be cleaned and repurposed for other patients, the PV should be treated like other personal items and not used without the patient’s explicit consent. A ventilator-sharing system could be ethically supportable if a PV user cannot continue to use their ventilator and another patient would benefit in conditions of scarcity, but this system needs numerous safeguards to ensure that sharing is voluntary and that the PV does not remain in use so long that the PV user has delayed discharge as a result. If a patient’s needs are best served by having their own caregiver at the hospital, this should be accommodated to the extent possible.

### Scenario 4

A PV user could be admitted to the hospital and develop acute respiratory distress syndrome (ARDS) from COVID-19 or another condition. In this scenario, the medical team would recommend switching to a ventilator capable of providing increased support; namely, switching from a basic ventilator to a full-featured ventilator—with COVID-19, oxygenation or high-precise FiO_2_ is a central concern. Such a patient would be subject to the same triage process as anyone else *for the full-featured ventilator*, so they may or may not receive the needed ventilator in the end.

How likely this scenario is will depend on (1) whether the PV really cannot provide the needed support and (2) how close the hospital is to CSC. Long-term ventilator users often have specialized expertise in how to adjust their ventilators for changing needs, so healthcare professionals should partner with these patients and share their own expertise as much as possible early in admission. If the triage criteria are such that the patient might not receive the full-featured ventilator, this may or may not be equitable allocation depending on the criteria and one’s conception of equity, which is unfortunately outside the scope of this paper.

## Triage Allocations and Disability Justice

As physicians and scientists have more time and data points to understand the trajectory of the novel coronavirus, they may determine that they can provide more and better care without relying so heavily on ventilators. Although less-invasive methods such as BiPAP (Bilevel Positive Airway Pressure) and CPAP (Continuous Positive Airway Pressure) may aerosolize secretions and pose risk to healthcare workers and other patients, high-flow nasal cannula could be as effective as intubation and mechanical ventilation yet without the many harmful side effects from long-term intubation.

As relevant as those facts are, the import of our analysis holds even if it is determined that ventilator use is ineffective for the COIVD-19 pandemic. Our arguments that the practice of PVR should be denied in all cases do not turn on the effectiveness of ventilators in CSC, but upon the ethical considerations of the experience of PV use. Furthermore, we have not provided an argument deduced from principles—whether nonmaleficence, beneficence, efficiency, efficacy, the public good, or what have you—but instead, an argument from the moral claims that arise from how people experience their lives.

We take the further implications of our arguments to extend to at least the following two claims. First, insofar as healthcare systems aim to both form a productive partnership with disability communities and also aim to deliver just and equitable care for patient populations at large, decision-making and deliberation concerning both policies and day-to-day clinical dilemmas should involve disability perspectives. Ideally, this should come from people inside of disability communities and, where that is impossible, disability allies and advocates. The integration of the perspective, experiences, and reflections of people with disabilities will *only improve* the ideal of just and equitable delivery of healthcare.^40^

Second, the moral distinction for which we have argued here, based as it is upon the lived experience of PV users, displays the broader analytic and clinical import of analysis grounded in first-person as opposed to merely third-person analysis. Third-person analyses arising from domains ranging from clinical biomedical research to quantitative sociology are profoundly valuable. We find, however, that they are significantly bolstered, especially with respect to ethical considerations, when supported and/or amended by first-person perspectives arising from phenomenological and other forms of qualitative analysis.

Furthermore, we contend that certain biomedical dilemmas, specifically with respect to their normative dimensions, will be poorly addressed except insofar as they are ultimately grounded in the latter and not the former. It is in light of these considerations that we argue that taking away someone’s PV is a direct assault on their bodily and social integrity, that PVs should not be part of reallocation pools, and that triage protocols should be immediately clarified and explicitly state that PVs will be protected in all cases.

## Notes

1.

Peterson A . Emily AL , Jason K . Ethics of reallocating ventilators in the Covid-19. BMJ 2020;369:m18283239822510.1136/bmj.m1828; Ballantyne A. ICU triage: How many lives or whose lives? *Journal of Medical Ethics Blog*; 7 April 2020; available at https://blogs.bmj.com/medical-ethics/2020/04/07/icu-triage-how-many-lives-or-whose-lives/ (last accessed April 2020); Stramondo J. Disability, likelihood of survival, and inefficiency amidst pandemic, *Bioethics.Net*; 6 April 2020; available at http://www.bioethics.net/2020/04/disability-likelihood-of-survival-and-inefficiency-amidst-pandemic/(last accessed April 2020); Schmidt H. Opinion | the way we ration ventilators is biased. *The New York Times*; 15 April 2020, sec. Opinion; available at https://www.nytimes.com/2020/04/15/opinion/covid-ventilator-rationing-blacks.html (last accessed April 2020).

2. Truog R, Christine M, George QD. The toughest triage—Allocating ventilators in a pandemic, *NEJM*; 23 March 2020; available at https://www.nejm.org/doi/full/10.1056/NEJMp2005689.

3. Food and Drug Administration, United States. *Enforcement Policy for Ventilators and Accessories and Other Respiratory Devices During the Coronavirus Disease 2019 (COVID-19) Public Health Emergency Guidance for Industry and Food and Drug Administration Staff*; 2020; available at https://www.fda.gov/media/136318/download.

4. Dowty D. Gov. Cuomo clarifies plan to seize ventilators, expects to get 500 statewide. *Syracuse*; 4 April 2020; available at https://www.syracuse.com/coronavirus/2020/04/gov-cuomo-clarifies-plan-to-seize-ventilators-expects-to-get-500-statewide.html (last accessed April 2020); French MJ. Cuomo plans to use national guard to seize ventilators from upstate facilities. *Politico PRO*; 3 April 2020; available at https://politi.co/39K6pHj (last accessed April 2020).

5. Faryon J. Nursing homes have thousands of ventilators that hospitals desperately need. *Kaiser Health News* (blog); 7 April 2020; available at https://khn.org/news/nursing-homes-have-thousands-of-ventilators-that-hospitals-desperately-need/ (last accessed April 2020).

6. Guidry-Grimes L, Savin K, Stramondo JA, Reynolds JM, Tsaplina M, Burke TB, et al. Disability rights as a necessary framework for crisis standards of care and the future of health care. The Hastings Center Report 2020;50(3);28–32. doi:10.1002/hast.112832596899.

7. Waserman D, Persad G, Millum J. Setting priorities fairly in response to Covid-19: Identifying overlapping consensus and reasonable disagreement. *Journal of Law and the Biosciences* 2020;7(1):1-12. doi:0.1093/jlb/lsaa044.

8. Wong A. Does the coronavirus pandemic make someone who is disabled like me expendable? *Vox*; 4 April 2020; available at https://www.vox.com/first-person/2020/4/4/21204261/coronavirus-covid-19-disabled-people-disabilities-triage?fbclid=IwAR3hVtKMShGV3Q6STEmHTLPbtYuU13SJ9EOwLQr9AryfZToCKdkFcOaZNQs (last accessed April 2020).

9. Mello MM, Persad G, White DB. Respecting disability rights—Toward improved crisis standards of care. *NEJM* 2020;383(5):e26. doi:056/NEJMp2011997.

10.

Altevogt BM , Stroud C , Hanson SL , eds. Institute of Medicine (US) Committee on Guidance for Establishing Standards of Care for Use in Disaster Situations. Washington, DC: National Academies Press; 2009; available at https://www.ncbi.nlm.nih.gov/books/NBK219958/. doi:10.17226/12749. See pg. 34.25032361

11. Ne’eman A. Opinion | ‘I will not apologize for my needs.’ *The New York Times*; 23 March 2020, sec. Opinion; available at https://www.nytimes.com/2020/03/23/opinion/coronavirus-ventilators-triage-disability.html (last accessed April 2020).

12. Fins JJ. New York state task force on life and the law ventilator allocation guidelines: How our views on disability evolved. *The Hastings Center*; 7 April 2020; available at https://www.thehastingscenter.org/new-york-state-task-force-on-life-and-the-law-ventilator-allocation-guidelines-how-our-views-on-disability-evolved/.

13. See note 9, Mello et al. 2020.

14. Idem.

15. See note 7, Wasserman et al. 2020.

16.

Galli G , Noel JP , Canzoneri E , Blanke O , Serino A . The wheelchair as a full-body tool extending the peripersonal space. Frontiers in Psychology 2015;6:9. doi:10.3389/fpsyg.2015.00639.26042069PMC4435246

17. Such a contention finds support in the growing research on embodied—and not just embedded—cognition. See Schmicking D , Gallagher S , eds. Handbook of Phenomenology and Cognitive Science. Dordrecht, Netherlands: Springer; 2010. doi:10.1007/978-90-481-2646-0.

18.

Preester H , Manos T . Body-extension versus body-incorporation: Is there a need for a body-model? Phenomenology and the Cognitive Sciences 2009;8(3):307–19. doi:10.1007/s11097-009-9121-y.

19. Stramondo JA. The distinction between curative and assistive technology. *Science and Engineering Ethics* 2019;25(4):1125-45. doi:10/gf9gcz.

20. Ibid. (1128).

21. Supporting this position, Havi Carel’s work on breathlessness further demonstrates that technologies concerning the lungs and respiratory system should not be treated similarly as, for example, a lower leg prosthesis or eyeglasses Carel H . Breathlessness: The rift between objective measurement and subjective experience. The Lancet Respiratory Medicine 2018;6(5):332–33. doi:10.1016/S2213-2600(18)30106-1;29523434 Williams T, Havi C. Breathlessness: From bodily symptom to existential experience. In Kevin A, ed. *Existential Medicine*. Wellcome Trust–Funded Monographs and Book Chapters. London, UK: Rowman & Littlefield International; 2018; available at http://www.ncbi.nlm.nih.gov/books/NBK535717/.

22. Aas S. Prosthetic embodiment. *Synthese* 2019. doi:10/ggwff7.

23. It cannot “dys-appear” in Drew Leder’s sense of the term. Leder D . The Absent Body. Chicago, IL: University of Chicago Press; 1990.

24.

Baynton D . Disability and the justification of inequality in American history In Longmore PK , Umansky L , eds. The New Disability History: American Perspectives. New York: New York University Press; 2001, at 33–57; Ordover N. *American Eugenics: Race, Queer Anatomy, and the Science of Nationalism*. Minneapolis: University of Minnesota Press; 2003.

25.

Kittay E . Deadly medicine: Project T4, mental disability, and racism. Research Philosophica 2016;93(4):715–41. doi:10.11612/resphil.1568.

26. Yancy G. Opinion | facing the fact of my death. *The New York Times*; 3 February 2020, sec. Opinion; available at https://www.nytimes.com/2020/02/03/opinion/death-religion-philosophy.html (last accessed April 2020).

27. Foggatt T. The MacGyvers taking on the ventilator shortage. *The New Yorker*.; 27 March 2020; available at https://www.newyorker.com/magazine/2020/04/06/the-macgyvers-taking-on-the-ventilator-shortage (last accessed April 2020).

28. Sanbern J. Eric Garner video: Staten island police chokehold death. *Time*; 23 July 2014; available at https://time.com/3016326/eric-garner-video-police-chokehold-death/ (last accessed April 2020).

29. CBS; 2020; available at https://minnesota.cbslocal.com/2020/05/26/george-floyd-man-dies-after-being-arrested-by-minneapolis-police-fbi-called-to-investigate/ (last accessed April 2020).

30.

Warraich H . Modern Death: How Medicine Changed the End of Life. New York, NY: St. Martin’s Press; 2017.

31. Truog R, Christine M, George QD. The toughest triage—Allocating ventilators in a pandemic. *NEJM*; 23 March 2020; available at https://www.nejm.org/doi/full/10.1056/NEJMp2005689.

32.

Sontag S . Illness as Metaphor and AIDS and Its Metaphors. New York, NY: Picador; 1977 https://us.macmillan.com/illnessasmetaphorandaidsanditsmetaphors/susansontag/9780312420130.

33. Hill C. ‘The cripples will save you’: Coronavirus message from a disability activist; 6 March 2020; available at https://creakyjoints.org/living-with-arthritis/coronavirus-disability-activism/ (last accessed April 2020).

34. See note 10, Altevogt et al. 2009.

35. Zucker H, Adler KP, Berens DP, Bleich JD, Brynner R, Butler KA, et al. *Ventilator Allocation Guidelines: New York State Task Force on Life and the Law New York State Department of Health*; 2015; available at https://www.health.ny.gov/regulations/task_force/reports_publications/docs/ventilator_guidelines.pdf.

36. See note 5, Faryon 2020; See note 1, Peterson et al. 2020; See note 12, Fins 2020.

37. See note 16, Galli et al. 2015.

38.

Fins JJ . Rights Come to Mind. Cambridge: Cambridge University Press; 2015; available at https://www.cambridge.org/core/books/rights-come-tomind/47C1316518BB222D79C7D5F6C8EED82A.

39. Ne’eman A. Do New York state’s ventilator allocation guidelines place chronic ventilator users at risk? Clarification needed. *The Hastings Center*; 3 April 2020; available at https://www.thehastingscenter.org/do-new-york-states-ventilator-allocation-guidelines-place-chronic-ventilator-users-at-risk-clarification-needed/.

40.

Garland-Thomson R. Human biodiversity conservation: A consensual ethical principle. The American Journal of Bioethics 2015;15(6):13–15; Citation omitted for review.10.1080/15265161.2015.102866326030486

