# Against Personal Ventilator Reallocation—ADDENDUM

**DOI:** 10.1017/S0963180120001103

**Published:** 2021-04

**Authors:** JOEL MICHAEL REYNOLDS, LAURA GUIDRY-GRIMES, KATIE SAVIN

In the article by Reynolds et al.^1^ in this issue, the following attribution was omitted from the acknowledgment:

The article was developed from earlier versions that appeared in the following blogs:

## Note

1.
Reynolds JM, Guidry-Grimes L, Savin K. forthcoming. Against personal ventilator reallocation. *Cambridge Quarterly of Healthcare Ethics* 2021;30(2). 10.1017/S0963180120000833.PMC764249933004101
